# Role of Genital Mycoplasmas in Bacteremia: Should We Be Routinely Culturing for These Organisms?

**DOI:** 10.1155/S106474499600066X

**Published:** 1996

**Authors:** Suzanne M. Garland, V. Nigel Kelly

**Affiliations:** Department of Microbiology and Infectious Diseases Royal Women's Hospital Carlton Victoria 3053 Australia

## Abstract

*Objective:* The purpose of this study was to examine the role of the genital mycoplasmas *Mycoplasma hominis* and *Ureaplasma urealyticum* as causes of bacteremia in a tertiary referral obstetrical, gynecological, and neonatal intensive care facility, over a period of 12 years from 1983 to 1994 inclusively.

*Methods:* All clinically significant blood cultures were reviewed and the percentage of septicemic episodes for genital mycoplasmas was compared to the total isolation rate, including conventional bacteria.

*Results:* The overall positivity rate for all pathogenic organisms isolated from the blood cultures of infants ranged from 4.5% to 7.7% per annum. *U. urealyticum* represented 0.8% of these positive isolates and *M. hominis* 0.4%. For adults, the overall positivity rate from blood cultures ranged from 6.5% to 13.5%, with *U. urealyticum* representing 9.6% of these positive isolates and *M. hominis* 9.9%.

*Conclusions:* With *M. hominis* having an established role in such clinical entities as postabortal and postpartum fever and *U. urealyticum* strongly implicated with chronic lung disease in low birth weight infants, it is appropriate to examine blood cultures for genital mycoplasmas in an obstetric institution.


Infectious Diseases in Obstetrics and Gynecology 4:329–332 (1996)
© 1997 Wiley-Liss, Inc.

### Role of Genital Mycoplasmas in Bacteremia: Should We Be Routinely Culturing for These Organisms?

**Suzanne M. Garland* and V. Nigel Kelly**
*Department of Microbiology and Infectious Diseases, Royal Women's Hospital, Carlton, Victoria, Australia*

### Abstract

***Objective:*** The purpose of this study was to examine the role of the genital mycoplasmas *Mycoplasma hominis* and *Ureaplasma urealyticum* as causes of bacteremia in a tertiary referral obstetrical, gynecological, and neonatal intensive care facility, over a period of 12 years from 1983 to 1994 inclusively.

***Methods:*** All clinically significant blood cultures were reviewed and the percentage of septicemic episodes for genital mycoplasmas was compared to the total isolation rate, including conventional bacteria.

***Results:*** The overall positivity rate for all pathogenic organisms isolated from the blood cultures of infants ranged from 4.5% to 7.7% per annum. *U. urealyticum* represented 0.8% of these positive isolates and *M. hominis* 0.4%. For adults, the overall positivity rate from blood cultures ranged from 6.5% to 13.5%, with *U. urealyticum* representing 9.6% of these positive isolates and *M. hominis* 9.9%.

***Conclusions:*** With *M. hominis* having an established role in such clinical entities as postabortal and postpartum fever and *U. urealyticum* strongly implicated with chronic lung disease in low birth weight infants, it is appropriate to examine blood cultures for genital mycoplasmas in an obstetric institution. Infect. Dis. Obstet. Gynecol. 4:329–332, 1996. © 1997 Wiley-Liss, Inc.

Key words

*Mycoplasma hominis; Ureaplasma urealyticum;* sepsis

The genital mycoplasmas *Mycoplasma hominis* and *Ureaplasma urealyticum* are commonly found as part of the endogenous microflora of the vagina of sexually active women, with colonization rates of 20% and 40–70%, respectively.[1,2] However, a clearer understanding of their role in disease has been made in the past decade. For example, it is now well recognized that *M. hominis* has an established role in pelvic inflammatory disease (PID), as researchers have not only isolated *M. hominis* in pure culture from Fallopian tubes of women diagnosed laparoscopically with PID, but have also documented a specific immune response to the organism.[2,3] Moreover, it has an established role in postabortal and postpartum fever,[2,4–6] pyelonephritis,[2,6–8] pneumonia, and meningitis in newborns,[2,9] while *U. urealyticum* not only has a role in preterm delivery, via chorioamnionitis, but also causes neonatal pneumonia and meningitis and is strongly associated with chronic lung disease in low birth weight infants.[2,10,11]

It is important to recognize that these potential pathogens have a limited spectrum of susceptibility to conventional antimicrobial agents. For example, they are both susceptible to tetracyclines, but not to the β-lactams, yet have differential susceptibility patterns to the macrolides and lincosamides. *U. urealyticum* is susceptible to erythromycin but not clindamycin, and the reverse is true for *M. hominis*.[11,12] Furthermore, these bacteria do not grow

*Correspondence to: Suzanne M. Garland, Department of Microbiology and Infectious Diseases, Royal Women's Hospital, Carlton, Victoria, Australia 3053.

Received 30 January 1996
Accepted 14 January 1997

TABLE 1. Significant blood culture isolates from an obstetrical, gynecological, and neonatal population from 1983 to 1994 inclusively

| | 1983 | 1984 | 1985 | 1986 | 1987 | 1988 | 1989 | 1990 | 1991 | 1992 | 1993 | 1994 | Average |
|---|---|---|---|---|---|---|---|---|---|---|---|---|---|
| Total population | | | | | | | | | | | | | |
| No. of isolates | 60 | 41 | 47 | 40 | 35 | 49 | 58 | 91 | 109 | 91 | 84 | 121 | 69 |
| Positivity rate—*U. urealyticum* (%) | 5.0 | 2.4 | 4.3 | 2.5 | 5.7 | 16.3 | 3.4 | 2.2 | 4.6 | 2.2 | 2.4 | 3.3 | 4.5 |
| Positivity rate—*M. hominis* (%) | 10.0 | 4.9 | 6.4 | 5.0 | 5.7 | 8.2 | 5.2 | 0.0 | 3.7 | 3.3 | 0.0 | 3.3 | 4.6 |
| Adults | | | | | | | | | | | | | |
| Total No. of isolates | 18 | 15 | 20 | 18 | 14 | 21 | 29 | 34 | 58 | 36 | 22 | 29 | 26 |
| *S. aureus* | 1 | 1 | 3 | 0 | 1 | 0 | 7 | 3 | 4 | 4 | 3 | 2 | |
| Streptococci | 0 | 0 | 1 | 4 | 4 | 1 | 2 | 4 | 15 | 7 | 2 | 7 | |
| Enteric gram-negative rods | 5 | 9 | 7 | 6 | 2 | 6 | 8 | 12 | 13 | 11 | 9 | 9 | |
| Anaerobic bacteria | 3 | 3 | 3 | 3 | 0 | 4 | 7 | 4 | 10 | 4 | 0 | 1 | |
| *U. urealyticum* | 2 | 1 | 1 | 1 | 2 | 7 | 1 | 2 | 5 | 2 | 2 | 4 | |
| *M. hominis* | 6 | 1 | 3 | 2 | 2 | 3 | 3 | 0 | 4 | 3 | 0 | 4 | |
| Other organisms | 1 | 0 | 2 | 2 | 3 | 0 | 1 | 9 | 7 | 5 | 6 | 2 | |
| Positivity rate—*U. urealyticum* (%) | 11.1 | 6.7 | 5.0 | 5.6 | 14.3 | 33.3 | 3.4 | 5.9 | 8.6 | 5.6 | 9.1 | 13.8 | 9.6 |
| Positivity rate—*M. hominis* (%) | 33.3 | 6.7 | 15.0 | 11.1 | 14.3 | 14.3 | 10.3 | 0.0 | 6.9 | 8.3 | 0.0 | 13.8 | 9.9 |
| Babies | | | | | | | | | | | | | |
| Total No. of isolates | 42 | 26 | 27 | 22 | 21 | 28 | 29 | 57 | 51 | 55 | 62 | 92 | 43 |
| *S. aureus* | 0 | 2 | 0 | 2 | 0 | 9 | 3 | 2 | 3 | 3 | 4 | 3 | |
| Coagulase-negative staphylococci | 2 | 4 | 9 | 6 | 4 | 3 | 9 | 16 | 21 | 30 | 40 | 60 | |
| Streptococci | 10 | 5 | 11 | 4 | 4 | 6 | 6 | 17 | 12 | 10 | 13 | 13 | |
| Enteric gram-negative rods | 21 | 9 | 6 | 5 | 10 | 3 | 9 | 9 | 6 | 6 | 1 | 9 | |
| Anaerobic bacteria | 3 | 4 | 0 | 4 | 2 | 2 | 1 | 6 | 5 | 0 | 0 | 3 | |
| *U. urealyticum* | 1 | 0 | 1 | 0 | 0 | 1 | 1 | 0 | 0 | 0 | 0 | 0 | |
| *M. hominis* | 0 | 1 | 0 | 0 | 0 | 1 | 0 | 0 | 0 | 0 | 0 | 0 | |
| Other organisms | 5 | 1 | 0 | 1 | 1 | 3 | 0 | 7 | 4 | 6 | 4 | 4 | |
| Positivity rate—*U. urealyticum* (%) | 2.4 | 0.0 | 3.7 | 0.0 | 0.0 | 3.6 | 3.4 | 0.0 | 0.0 | 0.0 | 0.0 | 0.0 | 0.8 |
| Positivity rate—*M. hominis* (%) | 0.0 | 3.8 | 0.0 | 0.0 | 0.0 | 3.6 | 0.0 | 0.0 | 0.0 | 0.0 | 0.0 | 0.0 | 0.4 |

readily in standard media and require special culture media and laboratory conditions for their detection and identification in clinical samples such as blood, cerebrospinal fluid, and genital swabs.[5,11] In particular, blood culture media containing sodium polyanetholesulfonate (SPS) inhibit the growth of both of these bacteria.[5]

We reviewed *M. hominis* and *U. urealyticum* as causes of bacteremia in an obstetrical, gynecological, and neonatal setting and contrasted them with conventional pathogens isolated over a 12-year period.

### MATERIALS AND METHODS

The Royal Women's Hospital, Melbourne, is a tertiary care obstetrical, gynecological, and perinatal university hospital and the largest in the State of Victoria, Australia. There are approximately 8,000 deliveries per annum. The neonatal intensive care unit has 12 ventilator beds, while the special care nursery has 42 beds.

For the period 1983–1994 inclusively, we reviewed the percentage of blood cultures positive for the genital mycoplasmas *M. hominis* and *U. urealyticum* and compared these with conventional organisms recognized as causes of septicemia. For adults these included enteric gram-negative rods, *Staphylococcus aureus*, *Streptococcus sp.* (groups A, B, C, D, and G, *S. milleri*, *S. pneumoniae*, *S. salivaris*, *S. oralis*, and the viridans group), and *Enterococcus sp.* and additionally for neonates, coagulase-negative staphylococci, where the same species was in more than one bottle and in the clinical setting of sepsis. Those isolates which were considered contaminants (e.g., *P. acnes* and coagulase-negative staphylococci) were excluded. This was not a population-based study for bacteremia; it was an assessment of positive blood cultures obtained from patients who were being screened on clinical grounds for septicemia. Indications for requesting blood cultures included for adults, clinical signs consistent with septicemia (persistent fever ≥38°C, with rigors ± hypotension) and laboratory indications of sepsis (e.g., leukocytosis with left shift). For neonates the indications included temperature instability, fever, persistent apneic episodes, bradycardia, acidosis, peripheral shutdown, or fitting.

TABLE 2. Clinical settings of significant blood culture isolates[a]

| Clinical setting | *S. aureus* | CoNS | *Streptococcus* sp. | Enteric GNB | Anaerobic bacteria | *U. urealyticum* | *M. hominis* | Other organisms[b] |
|---|---|---|---|---|---|---|---|---|
| Adults | | | | | | | | |
| Antepartum pyelonephritis | | | | ✓ | | | | |
| Sepsis postcesarean section | ✓ | | ✓ | | ✓ | ✓ | ✓ | |
| Sepsis postpartum | ✓ | | ✓ | | ✓ | | ✓ | ✓ |
| Sepsis abortion | ✓ | | | ✓ | ✓ | ✓ | | |
| Sepsis placenta privea | | | | | ✓ | | | |
| Chorioamnionitis | | | ✓ | ✓ | ✓ | ✓ | | ✓ |
| Sepsis gynecological oncology | | | ✓ | ✓ | ✓ | | | ✓ |
| Sepsis central venous line | ✓ | ✓ | | ✓ | | | | ✓ |
| Sepsis postvaginal hysterectomy | ✓ | | ✓ | ✓ | | ✓ | | |
| Postdilation and curettage | | | ✓ | | ✓ | | | |
| Post termination of pregnancy | | | | | | ✓ | | |
| Pelvic abscess | | | | | | | ✓ | |
| Ectopic pregnancy—salpingectomy | | | | | | ✓ | | |
| Pelvic infection | | | | | | | ✓ | |
| Pneumonia | | | ✓ | | | | | |
| Meningitis | | | ✓ | | | | | |
| Babies | | | | | | | | |
| Sepis, skin pustules | ✓ | | | | | | | |
| Comphalitis | ✓ | | | | | | | |
| Urinary tract infection | | ✓ | | ✓ | | | | |
| Necrotizing enterocolitis | ✓ | ✓ | | ✓ | ✓ | | | |
| Meningitis | | | ✓ | ✓ | | ✓ | ✓ | ✓ |
| Early-onset sepsis | | | ✓ | | ✓ | | | |
| Late-onset sepsis | ✓ | ✓ | ✓ | ✓ | | | | |

[a]CoNS, coagulase-negative *Staphylococcus* sp.; GNB, gram-negative bacteremia.
[b]Includes *Candida albicans, L monocytogenes, Pseudomonas* sp., *Hemophilus* sp., *Gardnerella vaginalis, C. jejuni, N. mucosa.*

From 1983 to 1986, the routine blood culture media used were an anaerobic Schaedler broth and an aerobic biphasic tryptone soya agar with 50 ml of tryptone soya broth medium, free of SPS, as previously described.[4] Media were incubated at 37°C for 7 days and inspected daily. Subculture of observed growth, as well as blind subculture at 48 h were performed as previously described, including culture for conventional aerobic and anaerobic bacteria and also for genital mycoplasmas using ureaplasma differential agar (modified A7) from the SPS-free aerobic medium.[4] Subsequently, in 1987, a change was made to the Bactec blood culture system (Becton, Dickinson & Co., Cockeysville, MD). The media used for adults were 6B (aerobic enriched tryptic soya broth) and 7DX (anaerobic enriched tryptic soya broth without SPS). For infants the media used were PedsPlus (enriched tryptic soya broth with antimicrobial removing resins) and 7DX. All Bactec media were incubated for 7 days at 37°C. The growth index was measured using the Bactec reader 460. Blind subculture was made from the SPS-free 7DX medium routinely after 48 h onto a modified A7 agar and incubated anaerobically for 2 days. Identification of *M. hominis* and *U. urealyticum* was as previously described.[4,5]

### RESULTS

*U. urealyticum* and *M. hominis* as a percentage of total significant blood culture isolates for the total population, with a breakdown into adults and infants, are shown in Table 1. The number of blood culture sets per year for the 12-year study period ranged from 622 to 1,196 for infants and 365 to 542 for adults. Overall the positivity rates for all pathogenic organisms for infants ranged from 4.5% to 7.7% and for adults 6.5% to 13.5%. *U. urealyticum* represented 0.8% of these positive isolates for infants and 9.6% for adults. Similarly, *M. hominis* represented 0.4% of these positive isolates for infants and 9.9% for adults.

The clinical context for adult cases included postabortal sepsis, postpartum endometritis, chorioamnionitis, fever plus retained products of conception, postpartum fever and pelvic collection, fever postremoval of intrauterine device, pelvic infection, and fever postdilatation and curettage. For infants, the clinical context included sepsis, meningitis, and investigation following premature delivery.

Conventional pathogens were isolated in a diverse array of clinical settings and are detailed in Table 2. In 9 instances, *M. hominis* or *U. urealyticum* was isolated in mixed culture. On 4 occasions they were found together in the clinical setting of postpartum sepsis. *M. hominis* was also detected in combination with *Gardnerella vaginalis* (chorioamnionitis), *S. aureus* (infected cesarean section wound), and polymicrobial anaerobic bacteria (postpartum sepsis). *U. urealyticum* was detected with *Klebsiella pneumoniae* (infected uterine cancer) and *G. vaginalis* (cuff cellulitis, postvaginal hysterectomy) separately.

### CONCLUSIONS

For adults it can be seen that genital mycoplasmas can contribute significantly to sepsis in an obstetric and gynecological setting. While these organisms are not highly pathogenic (they are not associated with septic shock as is the case with gram-negative bacillary sepsis), they contribute to increased bed stay and costs associated with the investigation of fever postpartum or postgynecological procedure. Moreover, they are important to specifically recognize as pathogens to ensure that appropriate antimicrobial treatment is utilized, especially where upper genital tract infection has occurred. Conventional antibiotic regimens for PID with β-lactams are ineffective against these microorganisms. Although in this review *M. hominis* and *U. urealyticum* did not represent as high a proportion of positive culture in infants compared to adult women, in the compromised host setting of the preterm infant, these organisms are important to recognize from normally sterile sites. *U. urealyticum* has been shown to cause meningitis[10,11] as well as being strongly implicated in chronic lung disease of low birth weight infants.[11,13,14] In conclusion, we believe that in a tertiary care obstetric, gynecological, and neonatal intensive care facility it is worthwhile and appropriate that the servicing microbiology laboratory specifically examine blood culture for the genital mycoplasmas in clinical settings where mycoplasmaemia is suspected.

### REFERENCES

1. McCormack WM, Rosner B, Alpert S, Evrard JR, Crockett VA, Zinner SH: Vaginal colonisation with *Mycoplasma hominis* and *Ureaplasma urealyticum*. Sex Transm Dis 134:67–70, 1986.
2. Cassell GH, Waites KB, Taylor-Robinson D: Genital mycoplasmas. In Morse SA, Moreland AA, Thompson SE (eds): Atlas of Sexually Transmitted Diseases. New York: Gower Medical Publishers, 7.2–7.15, 1990.
3. Stacey CM, Munday PE, Taylor-Robinson D, Thomas BJ, Gilchrist C, Ruck F, Ison CA, Beard RW: A longitudinal study of pelvic inflammatory disease. Br J Obstet Gynaecol 99:994–999, 1992.
4. Plummer DC, Garland SM, Gilbert GL: Bacteraemia and pelvic infection in women due to *Ureaplasma urealyticum* and *M. hominis*. Med J Aust 146:135–137, 1987.
5. Kelly VN, Garland SM, Gilbert GL: Isolation of genital mycoplasmas from the blood of neonates and women with pelvic infection using conventional SPS-free blood culture media. Pathology 19:277–280, 1987.
6. Taylor-Robinson D, McCormack WM: The genital mycoplasmas. N Engl J Med 302:1003–1010, 1063–1067, 1980.
7. Erno H, Thomsen AC: Immunoglobulin classes of urinary and serum antibodies in mycoplasmal pyelonephritis. Acta Pathol Microbiol Scand 88:237–240, 1980.
8. Thomsen AC: Occurrence and pathogenicity of *Mycoplasma hominis* in upper urinary tract: A review. Sex Transm Dis 10(4 Suppl):323–326, 1983.
9. Mardh PA: *Mycoplasma hominis* infection of the central nervous system in newborn infants. Sex Transm Dis 10(4 Suppl):331–334, 1983.
10. Garland SM, Murton LJ: Neonatal meningitis caused by *Ureaplasma urealyticum* (first case report). Paediatr Infect Dis 6:868–870, 1987.
11. Cassell GH, Waites KB, Watson HL, Crouse DT, Harasawa R: *Ureaplasma urealyticum* intrauterine infection: Role in prematurity and disease in newborns. Clin Microbiol Rev 6:69–87, 1993.
12. Senterfit LB: Antibiotic susceptibility testing of mycoplasmas. In Tully JG, Razin S (eds): Methods in Mycoplasmology. Vol 2. New York: Academic Press, pp 397–401, 1983.
13. Cassell GH, Waites DK, Crouse DT, et al: Association of *Ureaplasma urealyticum* infection of the lower respiratory tract with chronic lung disease and death in very low birth weight infants. Lancet 2:240–244, 1988.
14. Garland SM, Bowman ED: Role of *Ureaplasma urealyticum* and *Chlamydia trachomatis* in lung disease in low birth weight infants. Pathology (in press).
